# Evolution of the *Colocasiomyia gigantea* Species Group (Diptera: Drosophilidae): Phylogeny, Biogeography and Shift of Host Use

**DOI:** 10.3390/insects13070647

**Published:** 2022-07-18

**Authors:** Ling Xiao, Nan-Nan Li, Long-Kun Yang, Jia-Ling Li, Jian-Jun Gao

**Affiliations:** 1 Yunnan Key Laboratory of Plant Reproductive Adaptation and Evolutionary Ecology, Yunnan University, Kunming 650500, China; lingxiao@mail.ynu.edu.cn; 2 School of Forestry, Southwest Forestry University, Kunming 650224, China; li-nannan@foxmail.com (N.-N.L.); 1116021@aliyun.com (L.-K.Y.); 3 Wuzhishan Division, National Park of Hainan Tropical Rainforest, Wuzhishan 572215, China; 19071300110003@hainanu.edu.cn; 4 School of Ecology and Environmental Science, Yunnan University, Kunming 650500, China

**Keywords:** DNA sequence, geographical distribution, morphology, diversification, sister-group analysis, egg filament, body size, pollination mutualism, Monsteroideae, Araceae

## Abstract

**Simple Summary:**

All the species in the *Colocasiomyia gigantea* group breed on monsteroid host plants (aroids in the subfamily Monsteroideae). So far, we have not resolved the phylogenetic relationship among these fly species, making it difficult to trace the origin and history of the species diversification, biogeography and host plant selection. In this study, we reconstructed the evolutionary relationships between these species using multilocus DNA sequence data, and we inferred their ancestral areas and host plants. According to the results, this group diverged from its sister taxon through a split between the northeastern Oriental region and Sundaland + Wallacea, with the subsequent diversification occurring largely in the first region. We inferred the most likely ancestral host genus of this group to be *Rhaphidophora* Hassk, with possible subsequent shifts to *Scindapsus* Schott and/or *Epipremnum* Schott. We discuss the potential of the group as a model system for studies in evolutionary ecology and developmental genetics.

**Abstract:**

The *gigantea* species group of the genus *Colocasiomyia* de Meijere (Diptera: Drosophilidae) is among the four aroid-breeding species groups in this genus; however, it differs from the remaining three groups in the host use: all the flies in this group use plants from the subfamily Monsteroideae instead of from the subfamily Aroideae. So far, we have not resolved the phylogenetic relationship within this group, making it difficult to trace its geographical origin, pattern of species diversification and history of host plant use. In this study, we reconstructed the phylogenetic relationships within the *C. gigantea* group using DNA sequences of eight (two mitochondrial and six nuclear) gene markers, and we inferred the ancestral areas and host plants of the group based on the resulting phylogeny. According to the results, the *C. gigantea* group may have diverged from its sister group (i.e., the *C. cristata* group) through vicariance between the northeastern Oriental region and Sundaland + Wallacea, and the subsequent diversification of the *C. gigantea* group occurred mostly in the northeastern Oriental region, although an Oriental-to-Sundaland dispersal was followed by vicariance between these two areas, which finally gave rise to the *C. gigantea-C. scindapsae* lineage in the latter area. We inferred the most likely ancestral host plant of the *C. gigantea* group to be of the genus *Rhaphidophora* Hassk, with possible subsequent shifts to *Scindapsus* Schott and/or *Epipremnum* Schott plants. We discuss the potential for the egg filaments in the *C. gigantea* group to be used as a model system for comparative studies in pollination mutualism and developmental genetics concerning tubulogenesis.

## 1. Introduction

The genus *Colocasiomyia* de Meijere 1914 (Diptera, Drosophilidae) is an Old World pantropically distributed assemblage of 43 described and nearly 60 undescribed putatively new species, all feeding and breeding on the flowers of the lower angiosperms [1,2,3,4,5,6]. All these species were taxonomically assigned into six species groups, with each adapted to a specific host plant lineage: the *crassipes* group adapted to the family Magnoliaceae (the genus *Michelia* Linn.), the *zeylanica* group adapted to the family Arecaceae (the genus *Pinanga* Blume) and the remaining four groups adapted to the family Araceae: the *toshiokai*, *baechlii* and *cristata* groups adapted to the subfamily Aroideae, and the *gigantea* group adapted to the subfamily Monsteroideae (the genera *Rhaphidophora* Hassk, *Epipremnum* Schott and *Scindapsus* Schott, all belonging to the *Rhaphidophora* clade [7]) [3,4,5,8,9] (Table A1).

Since the 1980s, various researchers have investigated the phylogenetic relationships between the *Colocasiomyia* species via morphological comparison [1,2,3,4,10,11]. On the whole, a revised *Colocasiomyia* was supported as being monophyletic, with each of the four aroid-associated species groups (i.e., the *cristata*, *toshiokai*, *baechlii* and *gigantea* groups) and the *zeylanica* group of palm-breeding flies was supported. In a recent study [4], a cladistic analysis of 70 morphological characters of 24 species covering all six groups in *Colocasiomyia* was conducted, where the *gigantea* group (represented by *C. gigantea*, *C. scindapsae* and *C. rhaphidophorae*) was found to have the synapomorphy “foreleg tarsomere II elongated below”, and was placed as a sister to the *cristata* group. This synapomorphy in all the subsequently described species of the group, together with the appearance of egg filaments observed in the earliest member of the *gigantea* group, were confirmed in all the subsequently described species of the group, with the evolutionary significance of egg filaments readily recognized [4,5,8,9].

So far molecular phylogenetic analysis is still rare for *Colocasiomyia*, making it difficult to trace the evolutionary history of this genus. In a recent multilocus molecular phylogenetic analysis covering hundreds of species across the family Drosophilidae, a huge phylogenetic framework was constructed, in which the position of the genus *Colocasiomyia* was only poorly resolved. Among the three sampled species groups in this genus, i.e., *gigantea*, *toshiokai* and *cristata*, the former two were suggested with low confidence as closer to each other than either is to the third [12]. In the present study, we reconstructed the phylogenetic relationship in the *C. gigantea* group with DNA sequences of multiple gene loci and a full taxon sampling in the group. On the basis of this phylogenetic framework, we reconstructed and assessed the evolutionary history of this particular assemblage of species, paying special attention to the host use, biogeography and body sizes of the species.

## 2. Materials and Methods

### 2.1. DNA Markers, Taxon Sampling and DNA Sequencing

To reconstruct the phylogenetic relationship in the *gigantea* group, we used the same set of gene markers as in previous studies [6,13], including two mitochondrial genes (*COI* and *COII*: cytochrome *c* oxidase subunits I and II) and six nuclear genes (*28S rRNA*: 28S ribosomal RNA; *ATPsyn-alpha*: ATP synthases alpha gene; *ATPsyn-gamma*: ATP synthases gamma gene; *alphaTub84b*: alpha-Tublin at 84B; *Hsc70cb*: heat shock 70 kDa protein; *EF-2*: elongation factor 2) (Table 1). The corresponding sequences of five *gigantea* group species (i.e., *C. gigantea*, *C. hailini*, *C. longifilamentata*, *C. longivalva* and *C. scindapsae*), all used as outgroups in the abovementioned studies [6,13], were used again in this study, together with those of five species from the *cristata* group (i.e., *colocasiae*, *cristata*, *sarawakana*, *xenalocasiae* and *ecornuta*, representing the following lineages: clades I, IIa, IIb, IIc, and the independent branch leading to *ecornuta,* respectively), all of which were used as outgroup taxa considering their close morphological affinity to the *gigantea* group [4]. We did not consider the *toshiokai* groups, which was suggested as close to the *gigantea* group [12] since such a relationship was only weakly supported, as we discuss later in the Section 4.

We then determined the DNA sequences of the same set of markers for the remaining members of the *gigantea* group (i.e., *C. yini*, *C. rhaphidophorae*, *C. liae*, *C. todai* and *C. daiae*) (Table 1) using the same sets of PCR/sequencing primers as in a previous study [6], with adult specimens collected from host plants (or derived from eggs on host inflorescence) at varied sites in China (Table 1). We assembled the obtained trace files of the same target region in the SeqMan module of the DNAStar package version 7.1.0 (DNAStar Inc., Madison, WI, USA), with contigs made species by species, and manually examined and edited the ambiguities by eye. We then aligned the consensus sequences with the end nontarget regions trimmed, locus by locus, by using the CustalW tool in MEGA7 [14].

### 2.2. Data Partitioning and Model Selection

We concatenated the nucleotide sequences of all eight markers for the 15 sampled species, partitioned them into datasets by property (protein-coding gene: PCG vs. the *28S rRNA* gene), source (nuclear vs. mitochondrial), locus and codon position (Table S1). For each dataset (or dataset combination), nucleotide substitution model was selected in MEGA7. We then selected the optimal data-partitioning strategy among ten competing ones according to the Bayes factor (BF) criterion [15]. For this, we performed two parallel MCMC runs for 10^6^ generations (10^7^ generations for the strategy P_5_) in the analysis of each strategy, allowing the average standard deviation (STDEV) of the split frequencies to fall well below 0.01. We analyzed the resulting trace files in Tracer 1.7.1 [16].

### 2.3. Phylogenetic Reconstruction

Using RAxML HPC [17], starting from random trees, we calculated 20 distinct ML trees for the *gigantea* group under the selected optimal partitioning strategy and the GTR-GAMMA model. We used the five species in *cristata* as the outgroup taxa, evaluated the branch confidences using 1000 rapid bootstrap replications and drew bipartitions from the 1000 bootstrapped trees onto the best-scoring ML tree. A 50% majority rule consensus Bayesian tree was constructed in MrBayes v 3.2.6 [18] with the same partitioning strategy as in the ML analysis through two runs of 10^7^ MCMC generations. We generated a consensus tree after discarding the 20,000 initially sampled trees in either run.

### 2.4. Biogeographic Analyses

**Figure 1:**
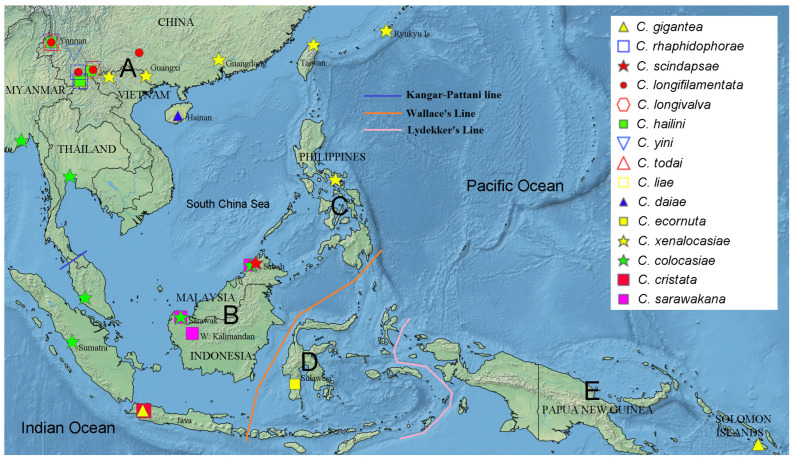
Division of the five areas (A: NE Oriental region, B: Sundaland, C: the Philippines, D: Wallacea, E: Australian region) employed in the biogeographical analysis. Records of geographic distributions of all known species in the *C. gigantea* group and five representative species of *C. cristata* group employed in the present study are shown with legends on the map (created with Simplemappr (http://www.simplemappr.net/; accessed on 28 May 2022).

We used RASP version 4 [19] to reconstruct the biogeographic history of the *gigantea* group through S-DIVA (statistical dispersal-vicariance analysis) on the ML tree constructed above. For this, we compiled data on the geographical ranges of the ten species in the *gigantea* group and the outgroup species from the literature [2,3,4,5,6,8,9], with the total range divided into five areas that referred to the Kangar-Pattani Line [20], Wallace’s Line [21] and Lydekker’s Line [21] (Figure 1): A—the northeastern Oriental region (referred to as the “NE Oriental region” below for convenience) with southern China, including Hainan and Taiwan + Ryukyu Islands + Sino-Himalaya + Indochina Peninsula; B—Sundaland; C—the Philippines; D—Wallacea; E—the Australian region (referred to as “Australian” below). Then, a S-DIVA analysis was conducted with four allowed areas in ancestral distribution.

### 2.5. Reconstruction of the Evolution of Host Use and Body Size

We reconstructed the evolution of the host use and body size in the *C. gigantea* group with Mesquite V3.70 [22] on the ML tree constructed in the present study. For this, we created a standard categorial matrix for the 15 representative *Colocasiomyia* species and two characters: the host use and body size. For the host use, we discriminated four states (Table 2) by referring to the literature [3,5,8,9] or our unpublished data: 0 (the subfamily Aroideae), 1 (the genus *Rhaphidophora*), 2 (the genus *Scindapsus*) and 3 (the genus *Epipremnum*).

For the body size, we used the thorax length (ThL) (defined as the distance from the anterior notal margin to the apex of the scutellum [23]), with data compiled from the original descriptions of the type specimens in the taxonomic literature or newly collected (Table A2), For this character, we discriminated two states (small vs. large): 0 (small: ThL ≤ 1.0 mm) and 1 (large: ThL ≥ 1.2 mm). For *Colocasiomyia liae*, only two specimen types were employed based on their original descriptions [8]. In our analysis, we treated the state of the body size of this species as ambiguous (indicated with “?” by default). We then reconstructed the likelihood of the ancestral states at the nodes of the ML tree. We traced the history of the two characters: the host use and body size. We then edited and combined the resulting charts in Adobe^®^ Photoshop^®^ CS6.

## 3. Results

### 3.1. DNA Sequencing, Model Selection and Data Partitioning

#### 3.1.1. DNA Sequencing

DNA sequences were newly determined for five species: *yini*, *rhaphidophorae*, *liae*, *todai* and *daiae* in the *C. gigantea* group (GenBank accession numbers: OM988102–106 for *COII*, OM943988–992 for *28S*, ON107163–167 for *ATPsyn-alpha*, ON107168–172 for *ATPsyn-gamma*, ON107158–162 for *alphaTub84B*, ON157517–521 for *Hsc70cb* and ON157522–526 for *EF-2*; Table S2). The alignment of the concatenated sequences of all these loci and 15 species spanned 4750 nucleotide sites, among which 1127 were variable and 649 were parsimony informative.

#### 3.1.2. Model Selection and Data Partitioning

The schemed DNA sequence data sets and respective selected models are shown in Table S1. Among the competitive partitioning strategies (Table 3), the strategy P_5_ (−ln*L* = 16,126.526) that making a distinction between the sources (mitochondrial vs. nuclear) and type (protein-coding or not) was selected as optimal by Bayes factor (Table 4).

### 3.2. Phylogenetic Relationship in the C. gigantea Group

**Figure 2:**
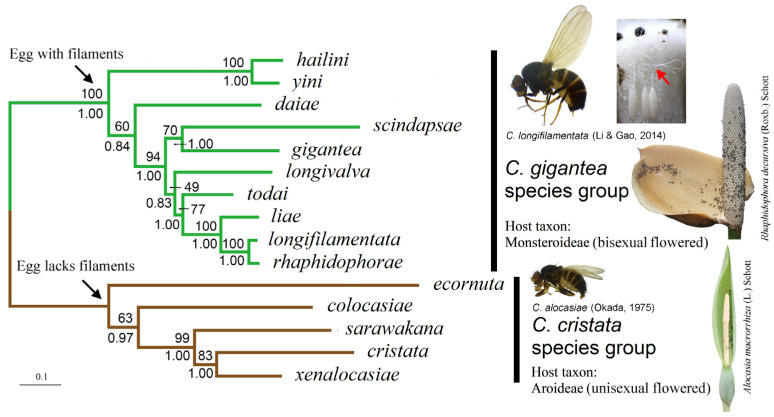
ML tree of ten species in the *C. gigantea* group (rooted with *C. cristata* group species). The tree was constructed with concatenated DNA sequences of eight gene loci and the data-partitioning strategy P_5_. Numbers beside nodes are bootstrap percentages and Bayesian posterior probabilities, respectively. Adults of *C. longifilamentata* and *C. alocasiae* are shown along with inflorescences of respective common host plants. Eggs of *C. longifilamentata* (all attached to the side wall of a pistil of *R. decursiva*) are also shown, with their filaments indicated with the red arrowhead.

We present the ML tree of the *C. gigantea* group built with the concatenated DNA sequences of eight gene loci and the data-partitioning strategy P_5_ in Figure 2. The tree strongly supported (we considered any bootstrap percentage (BP) above 75% and/or a posterior probability (PP) above 0.95 as strong support) the mutually monophyletic relationship between the *C. gigantea* and *C. cristata* groups (BP = 100; PP = 1.00).

Within the *gigantea* group, the “*C. hailini* + *C. yini*” clade was strongly supported (BP = 100, PP = 1.00), with the remaining eight species forming a weakly supported species cluster (BP = 60, PP = 0.84). Within this cluster, *C. daiae* was assigned as a sister to the collection of the seven remaining species (BP = 94, PP = 1.00). These latter seven species were divided into two clusters: the first was of the Southeast Asian *C. gigantea* and *C. scindapsae* (BP = 70, PP = 1.00), and the second, which was only weakly supported (BP = 49, PP = 0.83), was of five Chinese species (*C. longivalva*, *C. todai*, *C. liae*, *C. longifilamentata* and *C. rhaphidophorae*). Within this latter cluster, the grouping of the four species *C. todai*, *C. liae*, *C. longifilamentata* and *C. rhaphidophorae* was strongly supported (BP = 77, PP = 1.00), with well-resolved relationships among the four.

Among the outgroup species (i.e., the *C. cristata* species group), the previously defined “clade II” (represented here by *C. sarawakana*, *C. cristata* and *C. xenalocasiae*) [6] was strongly supported with respect to “clade I” (represented by *C. colocasiae*) (BP = 99, PP = 1.00), although with a different subclade branching order: the “subclades IIa” (represented by *C. cristata*) and “subclade IIc” (represented by *C. xenalocasiae*) were closer to each other than either was to “subclade IIb” (represented by *C. sarawakana*) (BP = 83, PP = 1.00).

### 3.3. Biogeography 

**Figure 3:**
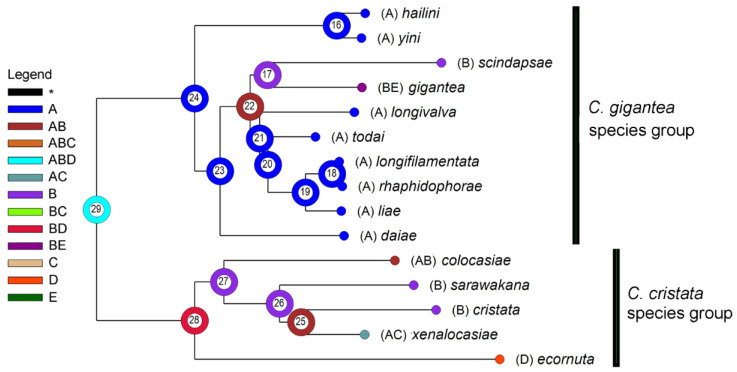
A reconstruction of ancestral areas (A: NE Oriental region; B: Sundaland; C: the Philippines; D: Wallacea; E: Australian region) in the *C. gigantea* group and the *C. cristata* group (out-group). The most likely state on each of the ancestral nodes (#16–29) is represented with a color/letter or color/letter combination. Distributions of extant fly species are mapped onto terminal nodes.

As shown in Figure 3 and Table 5, we inferred the range of the MRCA (most recent common ancestor) of the *gigantea* and *cristata* groups (node 29) as ABD (NE Oriental + Sundaland + Wallacea). Subsequent vicariance between A (NE Oriental) and BD (Sundaland + Wallacea) occurred then, and thereby gave rise to the MRCAs of the two groups. The MRCA of the *gigantea* group further split within A (node 24), and thereby gave rise to the *C. hailini*-*C. yini* lineage (node 16) and that of the remaining eight species (node 23). The former lineage split within A, and thereby gave rise to *C. hailini* and *C. yini*; the latter first dispersed to B and then split into two sublineages: one (restricted in A) finally gave rise to *C. daiae*, while the other (dispersed to B) gave rise to the MRCA of the remaining seven species (node 22). The further splitting of this latter sublineage between A and B gave rise to the MRCA of the *C. scindapsae*-*C. gigantea* pair in B and that of the NE Oriental pentad (*C. longivalva*, *C. todai*, *C. liae*, *C. longifilamentata* and *C. rhaphidophorae*) through a vicariance between A and B, with all the relevant speciation occurring within the respective area, except for *C. gigantea*, in which a further B-to-E dispersal (probably through D) occurred.

The ancestral area of the *C. cristata* group was reconstructed as BD (Sundaland + Wallacea), and it split into two sublineages through a vicariance between the areas B and D. The subsequent evolution may have involved a dispersal from Sundaland to the NE Oriental region (e.g., *C. colocasiae*) or the Philippines (e.g., *C. xenalocasiae*), and a vicariance between Sundaland and the NE Oriental region-the Philippines (*C. sarawakana* vs. *C. xenalocasiae*).

### 3.4. Ancestral Host Use and Evolution of Body Size

**Figure 4:**
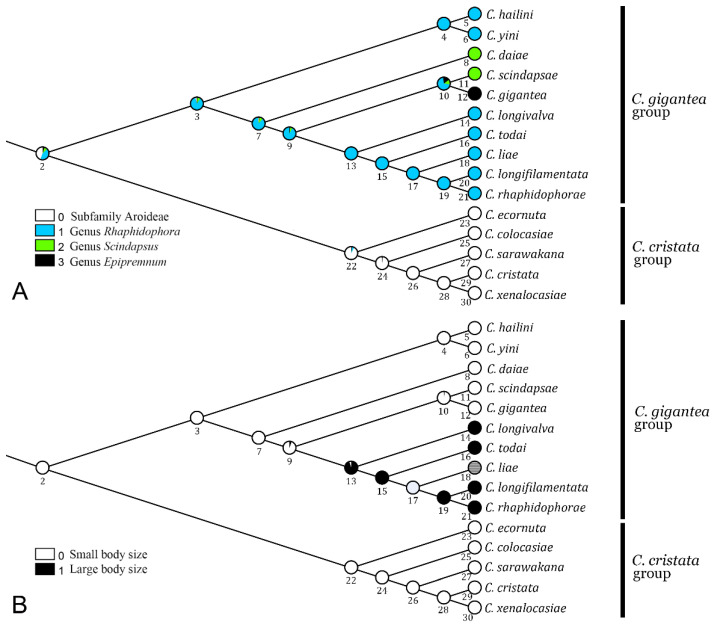
Evolutionary scenarios in (**A**) host plant use and (**B**) body size in the *C. gigantea* and *C. cristata* species groups. See Table A2 for data on body size.

As shown in Figure 4A, the host use was ambiguously reconstructed for the MRCA of the *C.* *gigantea* and *C. cristata* groups, either from Monsteroideae (*Rhaphidophora*, or much less likely, *Scindapsus* or *Epipremnum*) or from the subfamily Aroideae. We inferred with confidence that the MRCA of the *C. gigantea* group (node 3) used *Rhaphidophora* as a host. Although most of its descendant species retained the host selection of *Rhaphidophora*, two independent shifts to *Scindapsus* occurred: one to *C. daiae* and the other to *C. scindapsae*. For *C. gigantea*, a *Rhaphidophora*-to-*Epipremnum* shift occurred.

We inferred that the body size was ancestrally small in either the *gigantea* or the *cristata* group (Figure 4B). On the one hand, in the former group, the “large” body size may have evolved once in the MRCA of *C. longivalva*, *C. todai*, *C. liae*, *C. longifilamentata* and *C. rhaphidophorae*, even though in *C. liae*, the status was undetermined due to the deficiency of the type specimens [8]. On the other hand, for the *cristata* group, the body size was ancestrally “small”, which prevailed during the subsequent evolution of the group.

## 4. Discussion

### 4.1. Phylogeny of the C. gigantea Species Group

#### 4.1.1. Monophyly of the *Gigantea* Group and Its Relationship to the *Cristata* Group

In the recent cladistic analyses of 70 morphological characters and 24 *Colocasiomyia* species covering all six species groups of this genus [4], the *gigantea* and *cristata* groups were supported as reciprocally monophyletic, with both forming the most derived clade in the genus. The autapomorphy that supported the monophyly of the *gigantea* group (i.e., the bilateral lobes of the oviscapt (corresponding to the “hypogynium” in the standardized nomenclature of *Drosophila melanogaster* [24])) was “fused to each other only apically” (vs. “not fused” or “fused submedially to subapically” in the other groups) and was affirmed in all the subsequently replenished members in the *C. gigantea* group, except for *C. daiae;* in this latter species, the lateral lobes of the oviscapt were found fused to each other “only subapically” [9]. This feature, together with some other morphological features of the species (e.g., lobes of oviscapt lack warts on basal half, thorax with an additional pair of dorsocentral setae, wing costa with extraordinarily long setae and male abdominal sternite VI absence) [9], may be attributable to some forms of evolutionary adaptation.

#### 4.1.2. Phylogenetic Position of the *C. hailini*-*C. yini* Lineage

The pairing of these two species is consistent with the overall morphological similarity between them [5]. This pair was also recognized in the family-wide molecular tree [12], but was placed at a more or less derived position, forming a cluster with the Southeast Asian *C. gigantea,* and then with the cluster of *C. rhaphidophorae*, *C. longifilamentata* and *C. longivalva*. By closely examining the sequence dataset of this large-scale phylogenetic analysis [12], we found that it suffered from an overwhelming proportion of missing data. For example, ca. 81% of the schemed DNA sequences (272) were missing for the 16 *Colocasiomyia* species employed. Therefore, we relied on our results rather than those of the family-wide analysis. This was also why we chose to use the *cristata* group instead of the *toshiokai* group as the outgroup taxon in the present phylogenetic reconstruction.

#### 4.1.3. The *C. scindapsae*-*C. gigantea* Pair and *C. daiae*

In our phylogenetic tree, *C. daiae* branched right after the *C. hailini-C. yini* pair. It was likely that *C. daiae* retained “primordial” type of surstylus seen in the other two species: the surstylus was broad and ornamented, with three large peg-like prensisetae on the distal margin, with the lowest one distinctly elongated below [5,9].

#### 4.1.4. The *C. longivalva*-*C. todai*-*C. liae*-*C. longifilamentata*-*C. rhaphidophorae* Pentad

Despite the relatively lower bootstrap/posterior probability support for the clustering of the five large-bodied species (*longivalva*, *todai*, *liae*, *longifilamentata* and *rhaphidophorae*) in our phylogenetic analyses, two lines of morphological evidence support this pentad: (1) a relatively narrower and/or smaller surstylus, bearing only thin setae and/or tiny teeth (broad, with stout pegs or teeth in the small-bodied species), and (2) the presence of an epandrial posterior lobe (absent in the small-bodied species). Moreover, among these five species, the *longifilamentata*-*rhaphidophorae* pair exhibit some extraordinary morphological similarities with each other (e.g., epandrial posterior lobe well-developed and narrowly prolongated, scabbard-like, apically inlaid with a large peg [4,5,8,9]).

### 4.2. Biogeography, Major Host Shift, Diversification and Body-Size Evolution

According to our biogeographical analysis, a divergence between the *C. gigantea* and *C. cristata* groups took place through vicariance between the NE Oriental region and Sundaland + Wallacea, with the subsequent diversification of the former group occurring mostly within the NE Oriental region, despite two exceptions: (1) a NE Oriental→Sundaland dispersal followed by vicariance between these two areas, giving rise to the lineage of the large-bodied pentad in the NE Oriental region and to that of the *C. scindapsae-C. gigantea* pair in Sundaland; (2) the split between *C. scindapsae* and *C. gigantea* within Sundaland. However, taking into consideration the possible bias in our past surveys of the species diversity and host use of the *C. gigantea* group toward the NE Oriental region, the above scenario of the evolutionary history of the *C. gigantea* group was likely far from exhaustive. The genus *Rhaphidophora*, as the ancestral host taxa of the *C. gigantea* group, consists of ca. 100 species geographically occupying tropical Asia, and even extend as far as West Africa and the western Pacific, with the eastern Himalayas (from northern India and Nepal to southwestern China) recognized as one of its “hotspots” for endemic species [25,26]. It is thus intriguing to explore the dynamics underlying the evolution of the large body size in the lineage of the pentad *C. longivalva*, *C. todai*, *C. liae*, *C. longifilamentata* and *C. rhaphidophorae*, along with its adaptation to *Rhaphidophora* host plants, referring to the convergent feeding habit that occurred in the *C. hailini-C. yini* lineage. The other two host genera of the flies in the *C. gigantea* group, *Scindapsus* and *Epipremnum*, although of inferior species diversity (35 and 15 species, respectively [27]), are largely geographically overlapped with each other and with *Rhaphidophora*: *Scindapsus* occurs from northeastern India to western Polynesia [28], while *Epipremnum* ranges from southern Japan to Australia, and from India to the Cook Islands [29]. A future investigation of a wider geographical range, especially the NE Oriental region, is necessary to reconstruct the overall scenario of the diversification of the genus *Colocasiomyia*, including the *C. gigantea* group.

### 4.3. Adaptation of the C. gigantea Group to Monsteroid Host Plants

So far, varied evolutionary adaptations of the flies in the *C. gigantea* group have been assumed to evolved along with the Aroideae-to-Monsteroideae host shift of this fly group [4]. One character that accords with the host adaptation in the *C. gigantea* group is the blade-like oviscapt lobes, which are advantageous for laying eggs deep in the slits between the pistils on the host inflorescence [4,5,8,9]. Such an egg-laying mode protects the larvae (quiescent in the eggshell and harbored in the inflorescence) from biotic (e.g., predatory/parasitic natural enemies) and abiotic (low temperature (e.g., for species inhabiting the north tropical zone), drought, UV, etc.) stresses during the longstanding fruit-ripening process of the host plant [4]. We now know that for the eggs in all the species of the *gigantea* group, there are two thin, long and tubulate filaments that are horizontally arranged at the posterior end of the egg [4,5,8,9]. The filaments are indispensable to the respiration of the eggs and the embryo within the egg capsule hiding in the narrow slits between the pistils of the host inflorescence because the air exchange through the chorion is largely arrested within the slits [4,30,31]. Because egg filaments are not seen in the other *Colocasiomyia*, we can reasonably assume a single gain of such an apparatus in the MRCA of the *gigantea* group based on the principle of parsimony [32].

To date, no species in *Colocasiomyia* has ever been involved in evolutionary dating, making it difficult to explore its evolution against a concrete historical background. Nevertheless, we can make a rough judgment in the evolutionary context of the whole family Drosophilidae, taking into consideration the following date estimates: (1) 40–80 Mya (million years ago) for the splitting between the two subfamilies of Drosophilidae (i.e., Drosophilinae (with *Colocasiomyia* inside) and Steganinae) based on fossil and biogeographical data [33,34,35], and (2) 95 Mya (Nauheimer et al.; unpublished data as cited in a previous study [36]) or 89.2 Mya (95% highest probability density: 86.4–97.0 Mya) [37] for the splitting between the plant subfamilies Monsteroideae and Aroideae. Thus, we can reasonably interpret the initial adaptation of the *C. gigantea* group to some monsteroid host plant (and specifically a plant in the *Rhaphidophora* clade [7]) as a pollinator shift [38,39,40].

The evolution of egg filaments is a critical transformation that mediated the overall adaptation of the *C. gigantea* group to monsteroid host plants, which differ from those in the Aroideae according to a series of defining features [4], each of which may have exerted an essential influence on the flies’ adaptation to the monsteroid host plants (Table A1): (1) the spadices that are composed of bisexual florets are spatially homogeneous (i.e., without zonation and lacking any appendix), and thus, may provide only a few edibles for the larvae of the *Colocasiomyia* flies until the maturation of the spadix itself; (2) it is unlikely that the mass of the withered stamens of these hosts is sufficient to feed the larvae of the *Colocasiomyia*, and neither is the amount of pollen; (3) the whole spathe of the inflorescence, which is borne basal to the inflorescence, may thus hinder the *Colocasiomyia* larvae derived from eggs laid in the upper portion of the spadix from exploiting the bract itself; (4) the spathe falls off as a whole from its joint with the spadix soon after anthesis, which provides merely a momentary shelter for the *Colocasiomyia* larvae from environmental stress (i.e., the floral chamber, as in *Alocasia odora* [4]); and (5) in the NE Oriental region, where the *C. gigantea* group may have originated and where the major diversification of the group may have occurred, none of the presently known monsteroid plants bloom throughout the year (www.iplant.cn/info/Araceae?t=foc; accessed on 1 June 2022), which makes it difficult for the flies to depend solely on the spathe and/or pollen there.

Besides the flies in the *C. gigantea* group, a Neotropical *Drosophila* species was also found using the monsteroid plant (i.e., *D. monsterae:* the adults of this species are attracted into the floral chamber of *Monstera lentii* and copulate there during the female phase of the inflorescences, and they “stay at the spadix base where fallen pollen grains accumulated and later departed with pollen on their bodies.”) [41,42]. This finding indicates a parallel or convergent adaptation to the monsteroid host plant between *Colocasiomyia* and *Drosophila* flies. Moreover, according to the original description [42], the adults of *D. monstera* are characterized by a “small slightly circular compound eye” and a “conspicuously broad gena”, both commonly seen in *Colocasiomyia* flies.

### 4.4. The Potential of the C. gigantea Group in Evolutionary/Developmental Studies

So far, pollination mutualism have been examined in many aroid-breeding *Colocasiomyia* species and confirmed in some *cristata* species: the flies pollinate for their host plants. In turn, the plant inflorescence/infructescence rewards the flies with edibles for adults (exudates, solid substance from stamens) and larvae (the decaying pulp of fruits and/or basal soft tissue of dehisced pistils); a site for mating and oviposition; and a site/shelter for developing embryos, quiescent larval, pupae and even adults. In almost all such systems of pollinating mutualism, phenotypical synchrony between the lifecycles of the flies and their host plants has been observed [3,6,43,44,45]. The genus *Colocasiomyia*, with each of its groups specialized on a particular host plant lineage, provides us with an ideal system to explore the subtle mechanisms that underlie the evolution of intimate and obligate pollination mutualism, putting it on par with the fig-fig wasp and yucca-yucca moth systems [44]. A sister-group analysis allows control of “much potential noise and confounding variation which might otherwise afflict the analysis”, which makes it possible to “compare like with like” [46]. Among the groups in *Colocasiomyia*, the *cristata* and *gigantea* groups are of particular importance considering their sister-group relationship and their impressive intergroup divergence, according to the morphological, ecological and behavioral attributes, along with the major host shift that occurred in the latter group [4].

The distribution and phylogenetic importance of egg filaments in other drosophilid lineages at length, have been examined at length, with the gains and losses of such a structure in confirmed in varied taxa (e.g., the Hawaiian *Drosophila* and *Scaptomyza*) [47,48]. Indeed, some researchers considered the reduction in or absence of egg filaments to be a general property of tropical flower-breeding drosophilid species [49]. Within *Colocasiomyia*, egg filaments present in all the species in the *gigantea* group, but absent in the remaining groups [4,5,8,9]. In the fields of genetics and developmental biology, it is confirmed that such filaments of the eggshell, which are also known as dorsal appendages (DAs), arise at the end of oogenesis in *Drosophila melanogaster*; the tubulogenesis of this structure provides an excellent system that serves as a link between the patterning and morphogenesis phases, with eggshells of different drosophilid species used as the ideal testing model [50,51,52]. The species in the genus *Colocasiomyia*, especially those in the *gigantea* and *cristata* groups, provide an ideal comparative system for this field.

## 5. Conclusions

The present phylogenetic analysis lends strong support to the monophyly of the *C. gigantea* group with respect to the *C. cristata* group, placing the *C. hailini*-*C. yini* lineage as most basal within the *C. gigantea* group. Within the focal group, the Southeast Asian *C. scindapsae*-*C. gigantea* pair is well recognized; the northeastern Oriental, large-bodied *C. longivalva*-*C. todai*-*C. liae*-*C. longifilamentata*-*C. rhaphidophorae* pentad is supported with either molecular or morphological evidences.

The *C. gigantea* group diverged from the *C. cristata* group through a vicariance between the northeastern Oriental region and Sundaland + Wallacea. The subsequent diversification of this group occurred mostly within the former region. An Oriental-to-Sundaland dispersal occurred subsequently, followed by a vicariance between these two areas, finally gave rise to the *C. gigantea-C. scindapsae* lineage in Southeast Asian.

The most likely ancestral host genus of the *C. gigantea* species group is *Rhaphidophora*, with subsequent shift to *Scindapsus* and/or *Epipremnum* plants may had occurred. 

Within the whole genus *Colocasiomyia*, egg filaments (dorsal appendages) evolved exclusively in the *C. gigantea* group, investing the flies in this group with potential as ideal model system for comparative studies of pollination mutualism and developmental genetics concerning tubulogenesis.

## Figures and Tables

**Table 1:** Taxon sampling (and source of DNA sequences) in the present phylogenetic reconstruction.

Species Group	Species	Voucher #/Source of Sequence or Collection Site *							
		COI	COII	28S	ATPsyn-Alpha	ATPsyn-Gamma	alphaTub84B	Hsc70cb	EF-2
*cristata*	*colocasiae*	#01274/a	#01272/a	#01272/a	#01272/a	#01272/a	#01272/a	#01274/a	#01272/a
	*cristata*	#01254/a	#01254/a	#01254/a	#01254/a	#01254/a	#0125/a	#01254/a	#01254/a
	*sarawakana*	#01265/a	#01232/a	#01233/a	#01265/a	#01265/a	n/a	#01233/a	#01264/a
	*ecornuta*	#01267/a	#01267/a	#01267/a	#01267/a	#01267/a	#01267/a	#01267/a	#01267/a
	*xenalocasiae*	#01352/b	#01352/a	#01352/a	#01627/a	#01357/a	#01358/a	#01627/a	#01628/a
*gigantea*	*gigantea*	#01442/a	#01442/a	#01442/a	#01442/a	#01442/a	#01442/a	#01442/a	#01442/a
	*hailini*	#01517/b	#01448/a	#01517/a	#01448/a	#01448/a	#01448/a	#01448/a	#01448/a
	*longifilamentata*	#01252/a	#01133/a	#01134/a	#01134/a	#01134/a	#01134/a	#01134/a	#01134/a
	*longivalva*	#01722/a	#01722/a	#01722/a	#01722/a	#01722/a	#01722/a	#01722/a	#01722/a
	*scindapsae*	#01236/b	#01236/a	#01236/a	#01186/a	#01186/a	#01186/a	#01186/a	#01186/a
	*yini*	#00160/b	#01302/e	#01302/e	#01302/e	#01302/e	#01302/e	#01302/e	#01302/e
	*rhaphidophorae*	#01137/b	#01137/f	#01137/f	#01137/f	#01137/f	#01137/f	#01137/f	#01137/f
	*liae*	#10486/c	#12411/g	#12411/g	#12411/g	#12411/g	#12411/g	#12411/g	#12412/g
	*todai*	#10130/c	#09241/h	#09243/h	#09241/h	#09241/h	#09243/h	#09241/h	#09243/h
	*daiae*	#10566/d	#10566/i	#10566/i	#10566/i	#10566/i	#10566/i	#10566/i	#10566/i

* a–d, GenBank sequences [6]; b, ditto [5]; c, ditto [8]; d, ditto [9]; e, female adult derived from an egg on the inflorescence of Rhaphidophora decursiva (collection data: Baihualing, Baoshan, Yunnan, China, ? October 2012); f, female adult collected from the inflorescence of *R. hookeri* using an insect aspirator (Xishuangbanna Tropical Botanical Garden, Chinese Academy of Sciences, 3 March 2011); g, male adult derived from the infructescence of *R*. sp. (Qiongzhong, Hainan, China, ? December 2020); h, male adults derived from the infructescence of *R. peepla* (Baihualing, Baoshan, Yunnan, China, 3 December 2018); i, male adult collected from the inflorescence of *Scindapsus maclurei* using an aspirator (Qiongzhong, Hainan, China, ? December 2020).

**Table 2:** Statuses of the distribution areas, host plant uses and body sizes of ten species in the *Colocasiomyia gigantea* group and five representative species from the *C. cristata* group.

Species Group	Species	Geographical Distribution ^a^	Host Use ^b^	Body Size ^c^	
*C. gigantea* group	*hailini* Li and Gao, 2014	A	1	A	
	*yini* Li and Gao, 2014	A	1	A	
	*daiae* Xue and Gao, 2022	A	2	B	
	*scindapsae* Fartyal and Toda, 2013	B	2	A	
	*gigantea* Okada, 1987	BE	3	A	
	*longivalva* Li and Gao, 2014	A	1	B	
	*todai* Jiao and Gao, 2020	A	1	B	
	*liae* Li and Gao, 2020	A	1	?	
	*longifilamentata* Li and Gao, 2014	A	1	B	
	*rhaphidophorae* Gao and Toda, 2013	A	1	A	
*C. cristata* group	*ecornuta* Toda and Takano, 2021	D	0	A	
	*colocasiae* (Duda 1924)	AB	0	A	
	*sarawakana* Toda and Yafuso, 2021	B	0	A	
	*cristata* de Meijere, 1914	B	0	A	
	*xenalocasiae* (Okada 1980)	AC	0	A	

^a^ A: NE Oriental region, B: Sundaland, C: the Philippines, D: Wallacea, E: Australian region; ^b^ 0: subfamily Aroideae, 1: *Rhaphidophora*, 2: *Scindapsus*, 3: *Epipremnum*; ^c^ A: small, B: large, ?: not available.

**Table 3:** Data-partitioning strategies and the mean-ln*L* and 95% credible interval of each strategy.

Partitioning Strategy (Composition) ^a^	-ln*L*			
	ESS ^b^	Mean	95% HPD ^c^ Lower	95% HPD Upper
P_1_ (all sequence-concatenated)	4788	17,485.032	17,493.729	17,476.583
P_2a_ (mt, nu)	2853	17,142.185	17,152.597	17,133.047
P_2b_ (all-PCG, 28S)	4035	17,422.944	17,432.948	17,413.465
P_3a_ (mt, nu-PCG, 28S)	2802	17,069.504	17,097.743	17,059.122
P_3b_ (all-PCG-CP1+2, all-PCG-CP3, 28S)	3700	16,728.193	16,379.127	16,717.484
P_4_ (all-PCG-CP1, all-PCG-CP2, all-PCG-CP3, 28S)	2196	16,594.812	16,607.046	16,583.448
P_5_ (mt-CP1+, mt-CP3, nu-PCG-CP1+2, nu-PCG-CP3, 28S)	1856	16,126.526	16,138.411	16,115.309
P_7a_ (mt, and 6 partitions each for a nu gene)	3467	17,381.246	17,391.044	17,372.103
P_7b_ (mt-CP1, mt-CP2, mt-CP3, nu-PCG-CP1, nu-PCG-CP2, nu-PCG-CP3, 28S)	3114	16,784.351	16,793.752	16,775.353
P_8_ (8 partitions each for a gene locus)	3204	17,349.354	17,359.400	17,340.058

^a^ Abbreviations: mt: mitochondrial; nu: nuclear; PCG: protein-coding gene; CP: codon position; ^b^ ESS: effective sample size; ^c^ HPD: highest posterior density.

**Table 4:** The 2ln BFs of comparisons between partitioning strategies (1 vs. 2). A positive value indicates evidence against the strategy 2.

Strategy 2	Strategy 1								
	P_8_	P_7b_	P_7a_	P_5_	P_4_	P_3b_	P_3a_	P_2b_	P_2a_
P_1_	271.365	1401.362	207.572	2717.012	1780.440	−32,913.674	−31,390.284	−34,430.744	−28,850.346
P_2a_	−414.338	715.688	−478.122	2031.318	1094.746	−34,285.062	−32,761.672	−35,802.132	
P_2b_	147.180	1277.186	83.369	2592.836	1656.264	−33,162.026	−31,638.636		
P_3a_	−505.700	624.306	−569.484	1939.956	1003.384	−34,467.786			
P_3b_	−1242.322	−112.316	−1306.106	1203.334	266.762				
P_4_	−1509.084	−379.078	−1572.868	936.572					
P_5_	−2445.656	−1315.650	−2509.440						
P_7a_	63.784	1193.790							
P_7b_	−1130.006								

**Table 5:** Summary of the results of the S-DIVA analysis of ancestral distribution in the *C. gigantea* species group.

Node	Most Likely State	Event			Event Route *	Probability
		Dispersal	Vicariance	Extinction		
16	A	0	0	0	A→A^A→A|A	1.0000
17	B	1	0	0	B→B^B→BE^B→B|BE	1.0000
18	A	0	0	0	A→A^A→A|A	1.0000
19	A	0	0	0	A→A^A→A|A	1.0000
20	A	0	0	0	A→A^A→A|A	1.0000
21	A	0	0	0	A→A^A→A|A	1.0000
22	AB	0	1	0	AB→A|B	1.0000
23	A	1	0	0	A→A^A→AB^A→A|AB	1.0000
24	A	0	0	0	A→A^A→A|A	1.0000
25	AB	1	1	0	AB→ABC→B|AC	0.3333
26	B	1	0	0	B→B^B→AB^B→B|AB	0.3333
27	B	1	0	0	B→B^B→AB^B→AB|B	1.0000
28	BD	0	1	0	BD→D|B	1.0000
29	ABD	0	1	0	ABD→BD|A	1.0000

* About the symbols: “→” is used to link successive steps and to indicate their sequences; “^” is used to indicate isolation between populations; “|” is used to indicate a speciation event, either between two areas or within the same area.

## Data Availability

Data available in a publicly accessible repository.

## Supplementary Materials

The following supporting information can be downloaded at https://www.mdpi.com/article/10.3390/insects13070647/s1. Table S1: Data sets of DNA sequences and corresponding selected model (using the BIC i.e., Bayesian information criterion). Table S2: GenBank accession numbers of DNA sequences of eight molecular markers employed in the present study.

## Appendix A

**Table A1:** Comparison between host taxa of four aroid-breeding species groups in *Colocasiomyia*.

Host Subfamily	Character Status			
	Aroideae			Monsteroideae
Host tribe/clade/genus [27,53]	Genera: *Homalomena* Schott (*Philodendron* clade) and *Aglaonema* Schott (tribe Aglaonemateae)	Tribe: Schismatoglottideae (genera: *Schismatoglottis* and *Piptospatha*)	*Colocasia* clade; genera *Colocasia*, *Alocasia*, *Leucocasia* and *Steudnera*	*Rhaphidophora* clade (*Rhaphidophora*, *Scindapsus*, *Epipremnum*)
Corresponding species group in *Colocasiomyia*	*C. toshiokai* group (6 known species) [45]	*C. baechlii* group (2 known + 37 undescribed species) [54]	*C. cristata* group (21 known + 14 undescribed species) [6]	*C. gigantea* group (10 known species) [4]
Flower sexuality [27,53,55]	Unisexual			Bisexual
Spadix zonation [27,53,55]	Female/male	Female/sterile or bare/male	Female/sterile/male	No zonation
Spadix appendix [27,53,55]	Absent	Absent	Well developed	Absent
Spathe shape [27,53,55]	Without floral chamber (*Aglomena*) or varied (*Homalomena*)	Without floral chamber	With floral chamber	Boat-shaped (without floral chamber)

**Table A2:** Data of body size (thorax length) [23] of 15 *Colocasiomyia* species compiled from the literature or as newly collected data.

Species Group	Species	Thorax Length (ThL)		Code
		*n* (♂/♀)	Range (♂/♀; in mm)	
*C. gigantea* group	*hailini* Li and Gao, 2014 [5]	11/10	(0.76–0.94)/(0.89–1.22)	A
	*yini* Li and Gao, 2014 [5]	6/5	(0.90–1.12)/(0.93–1.20)	A
	*daiae* Xue and Gao, 2022 [9]	6/5	(0.90–1.07)/(0.86–1.24)	A
	*scindapsae* Fartyal and Toda, 2013 [4]	10/10	(0.88–1.03)/(0.85–1.24)	A
	*gigantea* Okada, 1987 [11]	n/a	n/a	A
	*longivalva* Li and Gao, 2014 [5]	4/8	(1.18–1.42)/(1.26–1.47)	B
	*todai* Jiao and Gao, 2020 [8]	6/5	(1.56–1.72)/(1.49–1.73)	B
	*liae* Jiao and Gao, 2014 [8]	1/1	1.10/0.93	?
	*longifilamentata* Li and Gao, 2014 [5]	7/5	(1.17–1.37)/(1.33–1.47)	B
	*rhaphidophorae* Gao and Toda, 2013 [4]	7/13	(1.30–1.48)/(1.18–1.57)	B
*C. cristata* group	*ecornuta* Toda and Takano, 2021 [6]	1 (?)	ca. 0.80	A
	*colocasiae* (Duda, 1924) [6]	1 (?)	ca. 0.94	A
	*sarawakana* Toda and Yafuso, 2021 [6]	1 (?)	ca. 0.86	A
	*cristata* der Meijere, 1914 [6]	1 (?)	ca. 1.05	A
	*xenalocasiae* (Okada, 1980) *	10/10	(0.65–0.80)/(0.66–0.92)	A

* Newly collected data from specimens from Vietnam (Lot 201: Mot Tuc Vill., Vietnam, 20.x.2011, *ex*. inflorescence of *Colocasia esculenta*, #2).
