# Supplementary material for: Evolution of the Colocasiomyia gigantea Species Group (Diptera: Drosophilidae): Phylogeny, Biogeography and Shift of Host Use
Source: Insects. 2022 Jul 18;13(7):647. doi: 10.3390/insects13070647 (PMC9319340; doi:10.3390/insects13070647)
Supplement: Supplementary file 1 [file insects-13-00647-s001.zip › Table_S1.pdf]

Table S1. Data sets of DNA sequences and corresponding selected model (using the BIC, i.e., Bayesian information criterion).

Data set†	Model	#Param	BIC	AICc	ln*L*	*I*	*Γ*	R	Freq A	Freq T	Freq C	Freq G	A-T	A-C	A-G	T-A	T-C	T-G	C-A	C-T	C-G	G-A	G-T	G-C
COI	GTR+G+I	37	6514.6663	6248.8166	-3087.2647	0.5713	1.2216	2.9217	0.3022	0.3699	0.1643	0.1634	0.10	0.00	0.04	0.08	0.21	0.00	0.00	0.47	0.00	0.08	0.00	0.00
COII	GTR+G+I	37	6773.6941	6506.4738	-3216.0985	0.5633	0.8585	3.4115	0.3393	0.3980	0.1432	0.1195	0.07	0.00	0.02	0.06	0.20	0.00	0.00	0.56	0.01	0.06	0.00	0.01
28S	T92+G	30	4254.2247	4038.0347	-1988.9243	n/a	0.2504	1.8242	0.3436	0.3436	0.1564	0.1564	0.06	0.03	0.11	0.06	0.11	0.03	0.06	0.23	0.03	0.23	0.06	0.03
ATPsyn-alpha	K2+G	29	2392.8880	2196.3612	-1069.0470	n/a	0.0500	5.6637	0.2500	0.2500	0.2500	0.2500	0.02	0.02	0.21	0.02	0.21	0.02	0.02	0.21	0.02	0.21	0.02	0.02
ATPsyn-gamma	K2+G	29	3042.3500	2846.9499	-1394.3361	n/a	0.2087	3.5205	0.2500	0.2500	0.2500	0.2500	0.03	0.03	0.19	0.03	0.19	0.03	0.03	0.19	0.03	0.19	0.03	0.03
alphaTub84B	K2+G	29	4448.4560	4239.7974	-2090.8106	n/a	0.1446	2.9340	0.2500	0.2500	0.2500	0.2500	0.03	0.03	0.19	0.03	0.19	0.03	0.03	0.19	0.03	0.19	0.03	0.03
Hsc70cb	K2+G	29	4962.0847	4757.6297	-2349.7131	n/a	0.3564	2.6742	0.2500	0.2500	0.2500	0.2500	0.03	0.03	0.18	0.03	0.18	0.03	0.03	0.18	0.03	0.18	0.03	0.03
EF-2	K2+G	29	3515.3487	3314.3184	-1628.0447	n/a	0.2353	3.2800	0.2500	0.2500	0.2500	0.2500	0.03	0.03	0.19	0.03	0.19	0.03	0.03	0.19	0.03	0.19	0.03	0.03
mt	GTR+G+I	37	13037.8187	12745.4897	-6335.6745	0.5733	1.1048	2.9838	0.3211	0.3842	0.1536	0.1410	0.09	0.00	0.03	0.07	0.20	0.00	0.00	0.51	0.01	0.07	0.00	0.01
mt-CP$_1$	TN93+G+I	34	3083.7594	2852.6993	-1392.1705	0.5060	0.3019	13.9507	0.2998	0.2772	0.1664	0.2566	0.01	0.00	0.05	0.01	0.32	0.01	0.01	0.53	0.01	0.05	0.01	0.00
mt-CP$_2$	HKY+G	32	1803.3681	1585.9337	-760.8077	n/a	0.0500	1.2166	0.2031	0.4233	0.2250	0.1485	0.10	0.05	0.08	0.05	0.12	0.04	0.05	0.22	0.04	0.11	0.10	0.05
mt-CP$_3$	TN93+G	33	7399.6491	7175.3649	-3554.5136	n/a	0.4995	18.2974	0.4601	0.4522	0.0695	0.0181	0.00	0.00	0.01	0.00	0.09	0.00	0.00	0.57	0.00	0.31	0.00	0.00
mt-CP$_{1+2}$	TN93+G+I	34	4789.8675	4535.0889	-2233.4550	0.7521	0.4091	8.0511	0.2515	0.3502	0.1957	0.2026	0.02	0.01	0.07	0.01	0.26	0.01	0.01	0.47	0.01	0.08	0.02	0.01
nuPCG	TN93+G	33	17656.4032	17373.6438	-8653.7931	n/a	0.2236	3.1514	0.2244	0.2474	0.2684	0.2595	0.03	0.03	0.13	0.03	0.27	0.03	0.03	0.24	0.03	0.11	0.03	0.03
nuPCG$_1$	TN93+G+I	34	4049.5862	3795.7452	-1863.7807	0.7171	0.5808	2.9576	0.2583	0.1876	0.2032	0.3508	0.02	0.02	0.08	0.03	0.32	0.04	0.03	0.30	0.04	0.06	0.02	0.02
nuPCG$_2$	HKY	31	3092.3601	2860.9481	-1399.3973	n/a	n/a	0.2215	0.3202	0.2824	0.2503	0.1470	0.11	0.10	0.03	0.13	0.05	0.06	0.13	0.05	0.06	0.06	0.11	0.10
nuPCG$_3$	GTR+G+I	37	9911.0151	9634.7076	-4780.2454	0.0516	1.5922	3.6240	0.0951	0.2722	0.3514	0.2804	0.08	0.07	0.28	0.03	0.21	0.01	0.02	0.16	0.02	0.09	0.01	0.02
nuPCG$_{1+2}$	K2+G+I	30	7059.3710	6814.5307	-3377.2295	0.4750	0.0500	1.6207	0.2500	0.2500	0.2500	0.2500	0.05	0.05	0.15	0.05	0.15	0.05	0.05	0.15	0.05	0.15	0.05	0.05
all	GTR+G+I	37	35355.7542	35017.5456	-17471.7524	0.4890	0.6232	2.1653	0.2721	0.2988	0.2148	0.2141	0.10	0.02	0.08	0.09	0.22	0.01	0.02	0.30	0.02	0.10	0.01	0.02
all-PCG	GTR+G+I	37	31138.9177	30806.5228	-15366.2375	0.4945	0.6766	2.1870	0.2572	0.2939	0.2294	0.2193	0.11	0.02	0.08	0.10	0.23	0.01	0.02	0.29	0.02	0.09	0.01	0.02
all-PCG$_{CP1}$	TN93+G+I	34	7003.6996	6735.6870	-3333.7828	0.6750	0.5175	5.3406	0.2724	0.2181	0.1907	0.3188	0.02	0.01	0.08	0.02	0.33	0.02	0.02	0.38	0.02	0.07	0.02	0.01
all-PCG$_{CP2}$	HKY+G+I	33	4739.0861	4479.0034	-2206.4444	0.4863	0.0500	0.6455	0.2805	0.3303	0.2417	0.1475	0.10	0.07	0.06	0.08	0.10	0.04	0.08	0.13	0.04	0.11	0.10	0.07
all-PCG$_{CP3}$	GTR+G	36	18260.1297	17976.3003	-8952.0824	n/a	1.0529	2.0315	0.2189	0.3332	0.2558	0.1915	0.15	0.02	0.09	0.10	0.20	0.00	0.02	0.27	0.02	0.10	0.01	0.02
all-PCG$_{CP1+2}$	TN93+G+I	34	11745.1435	11453.5292	-5692.7343	0.8128	0.5142	3.2635	0.2764	0.2741	0.2162	0.2332	0.03	0.02	0.09	0.03	0.26	0.03	0.03	0.32	0.03	0.10	0.03	0.02

†Abbreviations: mt, mitochondrial; nu, nuclear; PCG, protein-coding gene; CP, codon position.
