# Supplementary material for: Evolution of the Colocasiomyia gigantea Species Group (Diptera: Drosophilidae): Phylogeny, Biogeography and Shift of Host Use
Source: Insects. 2022 Jul 18;13(7):647. doi: 10.3390/insects13070647 (PMC9319340; doi:10.3390/insects13070647)
Supplement: Supplementary file 1 [file insects-13-00647-s001.zip › Table_S2.pdf]

Table S2. GenBank accession numbers of DNA sequences of eight molecular markers employed in the present study.

Species group	Species	Voucher specimens and corresponding GenBank accession numbers							
		COI	COII	28S	ATPsyn-alpha	ATPsyn-gamma	alphaTub84B	Hsc70cb	EF-2
cristata	*colocasiae*	(OK382964)[a]	(OK377094)[a]	(OK393944)[a]	(OK377073)[a]	(OK442375)[a]	(OK442396)[a]	(OK442415)[a]	(OK442436)[a]
	cristata	(OK382965)[a]	(OK377095)[a]	(OK393945)[a]	(OK373074)[a]	(OK442376)[a]	(OK442397)[a]	(OK442416)[a]	(OK442437)[a]
	sarawakana	(OK382975)[a]	(OK377107)[a]	(OK393957)[a]	(OK377086)[a]	(OK442388)[a]	n/a	(OK442428)[a]	(OK442449)[a]
	ecornuta	(OK382976)[a]	(OK377108)[a]	(OK393958)[a]	(OK377087)[a]	(OK442389)[a]	(OK442408)[a]	(OK442429)[a]	(OK442450)[a]
	xenalocasiae	(KY404116)[b]	(OK377113)[a]	(OK393963)[a]	(OK377092)[a]	(OK442394)[a]	(OK442413)[a]	(OK442434)[a]	(OK442455)[a]
gigantea	*gigantea*	(OK382967)[a]	(OK377097)[a]	(OK393947)[a]	(OK377076)[a]	(OK442378)[a]	(OK442399)[a]	(OK442418)[a]	(OK442439)[a]
	hailini	(KJ700928)[c]	(OK377098)[a]	(OK393948)[a]	(OK377077)[a]	(OK442379)[a]	(OK442400)[a]	(OK442419)[a]	(OK442440)[a]
	longifilamentata	(OK382969)[a]	(OK377100)[a]	(OK393950)[a]	(OK377079)[a]	(OK442381)[a]	(OK442402)[a]	(OK442421)[a]	(OK442442)[a]
	longivalva	(OK382970)[a]	(OK377101)[a]	(OK393951)[a]	(OK377080)[a]	(OK442382)[a]	(OK442403)[a]	(OK442422)[a]	(OK442443)[a]
	scindapsae	(KJ700886)[c]	(OK377102)[a]	(OK393952)[a]	(OK377081)[a]	(OK442383)[a]	(OK442404)[a]	(OK442423)[a]	(OK442444)[a]
	yini	(KJ700929)[b]	(OM988106)[f]	(OM943992)[f]	(ON107166)[f]	(ON107172)[f]	(ON107162)[f]	(ON157521)[f]	(ON157525)[f]
	rhaphidophorae	(KJ700893)[c]	(OM988104)[f]	(OM943989)[f]	(ON107165)[f]	(ON107170)[f]	(ON107161)[f]	(ON157159)[f]	(ON157523)[f]
	liae	(MT916870)[d]	(OM988103)[f]	(OM943990)[f]	(ON107164)[f]	(ON107169)[f]	(ON107160)[f]	(ON157518)[f]	(ON157526)[f]
	todai	(MT916897)[d]	(OM988105)[f]	(OM943991)[f]	(ON107167)[f]	(ON107171)[f]	(ON107159)[f]	(ON157520)[f]	(ON157524)[f]
	daiae	(OM037606)[e]	(OM988102)[f]	(OM943988)[f]	(ON107163)[f]	(ON107168)[f]	(ON107158)[f]	(ON157517)[f]	(ON157522)[f]

[a] Sequences determined in Takano *et al* (2021); [b] Sequences determined in Li *et al.* (2014); [c] Sequences determined in Shi *et al.* (2019); [d] Sequences determined in Jiao *et al.* (2020); [e] Sequences determined in Xue *et al.* (2022); [f] Sequences determined in the present study
